# Obstetric-specific compared to general early warning system for predicting severe postpartum maternal morbidity

**DOI:** 10.17305/bb.2024.11679

**Published:** 2024-12-18

**Authors:** Neža Pezdirc, Tatjana Stopar Pintarič, Miha Lučovnik

**Affiliations:** 1 Department of Perinatology, Division of Obstetrics and Gynecology, University Medical Centre Ljubljana, Ljubljana, Slovenia; 2 Department of Anaesthesiology and Surgical Intensive Care, University Medical Centre Ljubljana, Ljubljana, Slovenia; 3 Faculty of Medicine, University of Ljubljana, Ljubljana, Slovenia

**Keywords:** Severe maternal morbidity, postpartum period, modified early obstetric warning system, MEOWS

## Abstract

Severe maternal morbidity is a major global health concern, and early identification of at-risk postpartum women is essential to improving outcomes. We aimed to compare the predictive values of the Modified Early Obstetric Warning System (MEOWS) versus the non-obstetric general early Warning System (EWS) for predicting severe maternal morbidity in postpartum women. We retrospectively reviewed hospital documentation of 723 postpartum women admitted to the obstetric high dependency unit between October 2020 and March 2021. Severe maternal morbidity was defined using the American College of Obstetricians and Gynecologists’ criteria. We assessed the sensitivity, specificity, positive and negative predictive values, as well as positive and negative likelihood ratios, of the MEOWS and the EWS for predicting severe postpartum maternal morbidity. Twenty-four (3.3%) women included in the study met the criteria for severe maternal morbidity. Hypertensive complications and obstetric haemorrhage were the most prevalent causes of maternal morbidity. The sensitivity of the MEOWS was 92%, specificity 62%, positive predictive value 8%, and negative predictive value 100%. The positive likelihood ratio was 2.4, while the negative likelihood ratio was 0.1. In comparison, the EWS had a sensitivity of 63%, specificity of 66%, positive predictive value of 6%, and negative predictive value of 98%. The positive likelihood ratio for the EWS was 1.8, and the negative likelihood ratio was 0.6. The obstetric-specific early warning system proved to be superior for the early prediction of severe postpartum maternal morbidity compared to the general non-obstetric warning system.

## Introduction

Early warning systems (EWSs) were developed to detect the deterioration of hospitalized patients early in the course of the disease and allow timely interventions to prevent complications by monitoring deviations from normal physiological parameters [1]. Numerous studies have demonstrated a reduction in in-hospital mortality following the implementation of EWS in non-pregnant patients [2]. During pregnancy, a woman’s body undergoes several physiological changes, such as increased blood volume, altered cardiovascular dynamics, and hormonal fluctuations, which can affect vital signs like heart rate, blood pressure, and respiratory rate. These changes may complicate the detection of abnormalities, making it essential to modify EWS for pregnant patients [3, 4].

The Modified Early Obstetric Warning System (MEOWS) was specifically designed for monitoring pregnant and postpartum women. However, it was developed empirically, through adjustments to the EWS scales, based on the expected normal physiological values pertinent to pregnant and postpartum women [5–8]. Several studies have reported the predictive value of MEOWS for the early detection of specific peripartum maternal complications, such as severe preeclampsia, complications related to postpartum hemorrhage, sepsis, renal impairment, amniotic fluid embolism, and the need for maternal admission to the intensive care unit [9–16]. Nevertheless, there is very limited evidence that MEOWS outperforms general EWS in predicting postpartum maternal morbidity [3, 4, 17].

The aim of this study was to directly compare the predictive values of MEOWS to those of non-obstetric general EWS in predicting severe morbidity in postpartum women.

## Materials and methods

### Study design and study population

This was a retrospective cohort study in which we reviewed the medical records of postpartum women admitted to the obstetric high-dependency unit (HDU) at the Department of Perinatology, University Medical Centre Ljubljana, Slovenia, between October 2020 and March 2021. All women who underwent cesarean delivery and all women experiencing complications during or after vaginal delivery are monitored in the HDU at our institution for at least 48 h after birth.

### Data collection

Physiological parameters are monitored regularly at specific time intervals after HDU admission and recorded on a temperature chart equipped with MEOWS.

The following nine parameters are routinely monitored as per the standard operating procedure:

- Respiratory rate
- Blood oxygen saturation
- Need for supplemental oxygen
- Body temperature
- Systolic and diastolic blood pressure
- Heart rate
- Level of consciousness, assessed using the AVPU alertness scale (an acronym for “alert, verbal, pain, unresponsive”)
- Pain, assessed using the visual analog scale (VAS)

Values are scored based on the MEOWS criteria, with scores ranging from 0 to 3 points (Table 1). Based on the total score, women can be classified into one of three MEOWS groups: red, yellow, or green. Medical staff then act according to the defined response system. Scores of 1–4 (green) should prompt informing the lead nurse/midwife and may trigger more intensive monitoring. More intensive monitoring (at least one measurement every 30 min) should be instituted with scores of 5–6 or at least one value of 3 points (yellow). A physician (anesthesiologist or obstetrician) should be notified at this point. For scores of ≥ 7 points or in the case of acute deterioration of the woman’s condition, continuous monitoring should be started, and senior anesthesiology and obstetric physicians should be called to the bedside.

**Table 1:** Threshold values of individual physiological parameters and their point evaluation according to the criteria of the Modified Early Obstetric Warning System

**Score**			
**Parameter**	**1**	**2**	**3**
Respiratory rate (min^−1^)		21–25	<12 or >25
Arterial blood oxygen saturation (%)		92–95	<92
Oxygen supplementation		yes	
Body temperature (^∘^C)		37.3–37.7	>37.7 or <36.0
Systolic blood pressure (mmHg)	140–150	150–160	>160 or <90
Diastolic blood pressure (mmHg)	90–100	100–110	>110
Heart rate (min^−1^)	110–120	120–130 or 50–60	>130 or <50
Conscious level (AVPU scale)			V or P or U
VAS pain score		4–7	≥7

AVPU: Alertness scale (an acronym from “alert, verbal, pain, unresponsive”); VAS: Visual analog scale for pain intensity assessment.

For the purpose of this study, we assessed all women included using the general EWS recommended for evaluating adult non-obstetric patients at our hospital simultaneously with MEOWS scoring. Table 2 presents the EWS scoring criteria. Nurse/midwifery staff should inform a medical doctor about all cases with a total EWS score of 2 or more points. When the EWS score exceeds 3 points, the hospital emergency medical service should be contacted.

**Table 2:** Threshold values of individual physiological parameters and their point evaluation according to the criteria of non-obstetric early warning system for adults

**Score**			
**Parameter**	**1**	**2**	**3**
Heart rate (min^−1^)	41–50 or 101–110	111–129 or <40	>130
Systolic blood pressure (mmHg)	81–100	71–80 or >200	<70
Respiratory rate (min^−1^)	15–20	21–29 or <8	>30
Temperature (^∘^C)	35.1–36.5 or >37.5	<35	
Conscious level (AVPU scale)	V	P	U

AVPU: Alertness scale (an acronym from “alert, verbal, pain, unresponsive”).

### Patient evaluation

#### Severe maternal morbidity definition

Several diagnostic criteria for severe maternal morbidity have been proposed in the literature [17–21]. In the present study, we defined severe postpartum maternal morbidity using criteria proposed by the American College of Obstetricians and Gynecologists (ACOG) [21].

### Ethical statement

The study was approved by the National Medical Ethics Committee of the Republic of Slovenia on July 30, 2021 (No. 0120-8/2021/6).

### Statistical analysis

Demographic and clinical characteristics of women with and without severe maternal morbidity were compared using the Student’s *t*-test or the Mann–Whitney *U* test (in cases of non-normal distribution). For categorical variables, the chi-squared test or Fisher’s exact test was used to compare the groups. A *P* value of < 0.05 was considered statistically significant.

Sensitivity, specificity, positive and negative predictive values, as well as positive and negative likelihood ratios for various MEOWS and EWS score groups in predicting severe maternal morbidity were calculated.

Data were analyzed using IBM SPSS Statistics Version 27 (IBM Corp., Amrok, NY, USA) and MedCalc Diagnostic Test Evaluation Calculator (MedCalc Software Ltd., Acacialaan, Belgium).

## Results

We included 723 women in the study. Twenty-four (3.3%) met the ACOG criteria for severe maternal morbidity. Among the causes of maternal morbidity, hypertensive disorders were the most common (13/24, 54.2%), followed by massive postpartum hemorrhage (8/24, 33.3%), pulmonary complications (2/24, 8.3%), and anesthesia complications (1/24, 4.2%).

A comparison of the demographic and clinical characteristics of women with and without severe postpartum maternal morbidity is presented in Table 3. Apart from the expected longer HDU stay, the groups only differed significantly in terms of gestational age at delivery. Women experiencing severe morbidity delivered at significantly earlier gestations (*P* < 0.001).

**Table 3:** Comparison between women with vs without severe maternal morbidity postpartum

	**Women without severe maternal morbidity (*n* ═ 699)**		**Women with severe maternal morbidity (*n* ═ 24)**		***P* value**
	**median/*n***	**Q_1_–Q_3_/%**	**median/*n***	**Q_1_–Q_3_/%**	
Age, years	32.0	28.0–35.0	30.5	28.0–33.5	0.241
Maternal pre-pregnancy BMI, kg/m^2^	23.9	21.4–27.5	23.6	21.3–28.8	0.918
Maternal BMI at delivery, kg/m^2^	29.2	26.4–32.7	30.1	25.9–35.7	0.713
Gestational age at delivery, weeks	39.1	38.0–40.0	36.8	31.5–38.5	<0.001
Nulliparity	367	52.0	17	70.8	0.096
Twin pregnancy	31	4.4	2	8.3	0.300
Vaginal delivery	117	16.7	5	20.8	0.080
Planned cesarean section	252	36.1	3	12.5	
Emergency cesarean section	321	45.9	16	66.7	
Operative vaginal delivery	9	1.3	0	0	
Postpartum hemorrhage before admission to the HDU, mL	400	300–500	500	300–1630	0.073
Days in the HDU	2	2–2 (1–11)	3	3–4 (2–10)	<0.001

Data are presented as median and interquartile range (Q_1_–Q_3_) or number of women (*n*) and percentage of women (%). *P* ≤ 0.05 is considered statistically significant. HDU: High dependency unit; Q_1_: 1^st^ quartile; Q_3_: 3^rd^ quartile; BMI: Body mass index; *P*: *P* value

Predictive values for different MEOWS and EWS score groups are shown in Table 4. When comparing the MEOWS yellow or red groups to the EWS ≥ 2 points group, both requiring informing physicians about the patient’s condition, MEOWS performed better than general EWS. Sensitivity was higher (92% [95% confidence interval (CI) 73%–99%] vs 63% [95% CI 41%–81%]) with only slightly lower specificity (62% [95% CI 58%–66%] vs 66% [95% CI 62%–69%]). Positive and negative predictive values were comparable but higher for MEOWS (8% [95% CI 7%–9%] vs 6% [95% CI 4%–8%] and 100% [95% CI 98%–100%] vs 98% [95% CI ═ 97%–99%]). Similarly, the positive likelihood ratio was higher (2.4 [95% CI 2.1–2.8] vs 1.8 [95% CI 1.3–2.5]) and the negative likelihood ratio was lower (0.1 [95% CI 0.04–0.5] vs 0.6 [95% CI 0.3–1.0]) for MEOWS compared to general EWS.

**Table 4:** Predictive values of different MEOWS groups and EWS groups for prediction of severe maternal morbidity in postpartum women

	**Sensitivity**	**Specificity**	**Positive predictive value**	**Negative predictive value**	**Positive likelihood ratio**	**Negative likelihood ratio**
MEOWS red or yellow	92% (73%–99%)	62% (58%–66%)	8% (7%–9%)	100% (98%–100%)	2.4 (2.1–2.8)	0.1 (0.04–0.5)
MEOWS red	58% (37%–78%)	99% (98%–100%)	70% (50%–85%)	99% (98%–99%)	68.0 (28.6–161.5)	0.4 (0.3–0.7)
MEOWS yellow	33% (16%–55%)	63% (59%–67%)	3% (2%–5%)	96% (95%–97%)	0.9 (0.5–1.6)	1.1 (0.8–1.4)
MEOWS green	8% (1%–27%)	58% (54%–62%)	0.7% (0.2%–2.5%)	95% (94%–95%)	0.2 (0.1–0.8)	1.6 (1.4–1.8)
EWS ≥ 2 points	63% (41%–81%)	66% (62%–69%)	6% (4%–8%)	98% (97%–99%)	1.8 (1.3–2.5)	0.6 (0.3–1.0)
EWS ≥ 4 points	8% (1%–27%)	99% (98%–100%)	25% (7%–61%)	97% (97%–97%)	9.7 (2.1–45.6)	0.9 (0.8–1.0)

Data are presented as values (95% confidence interval). Shaded rows highlight the validation results for comparable groups of the MEOWS (red or yellow group) and the EWS (EWS ≥ 2 points), both requiring informing physicians about the patient’s condition. MEOWS: Modified Early Obstetric Warning System; EWS: Early Warning System for adult non-obstetric patients; n: Number of women.

## Discussion

While it might seem obvious that an obstetric-specific EWS would outperform a non-specific EWS for predicting deterioration in obstetric patients, it is important to note that obstetric-specific scales have not been extensively validated [3, 4]. Furthermore, the diagnostic performance of early warning scoring systems adapted for the obstetric population has not been directly compared to non-specific early warning scales in the same group of pregnant or postpartum women [3].

Our study was the first to directly compare the predictive values of MEOWS to those of a general adult EWS for the prediction of postpartum maternal morbidity. The most clinically relevant measures of the predictive ability of the two warning systems are the positive and negative likelihood ratios. These measures indicate how much the likelihood of severe complications changes when the trigger criteria in each system are met [22]. In the context of our study, the likelihood ratio provides a measure of how much the odds of severe maternal morbidity change for a postpartum woman who meets the criteria of a specific MEOWS or EWS score group (positive likelihood ratio) or does not meet them (negative likelihood ratio). Importantly, likelihood ratios are not affected by the incidence of severe maternal morbidity in the study population [21]. In MEOWS validation studies published so far, likelihood ratios were not calculated [17–20]. Positive likelihood ratios for MEOWS groups that trigger escalation of care were higher than those of general EWS. On the other hand, negative likelihood ratios for MEOWS were lower or not significantly different from those of EWS. This means that MEOWS helps identify postpartum women at risk of serious complications more accurately without increasing unnecessary interventions and causing “alarm fatigue”.

The results of previous studies evaluating MEOWS for predicting severe maternal morbidity vary widely in terms of the population studied (pregnant vs postpartum women), hospital units where the research took place (different levels of care), and the diagnostic criteria used for defining severe maternal morbidity. These differences make it challenging to directly compare our study with the existing literature in the field. However, our findings are in line with most previous research on this topic. Singh et al. [18] and Singh et al. [19] reported similar accuracy of MEOWS in predicting severe maternal morbidity defined ad hoc by the authors in their studies. In our study, the calculated sensitivity of MEOWS was higher for identifying severe maternal morbidity using the established ACOG criteria. On the other hand, specificity and positive predictive value were lower. It is important to point out that for either EWS, the positive predictive value was relatively low compared to the negative predictive value. This suggests that patients who trigger an alert require further evaluation. Conversely, patients who do not trigger an alert are unlikely to have a serious, life-threatening condition. The negative predictive value of EWSs is, therefore, more clinically helpful than their positive predictive value.

The main limitation of our study is the small number of postpartum women with complications that met the severe maternal morbidity criteria. Although the study included a relatively large number of postpartum women, the final number of those with serious life-threatening complications was small due to the rarity of such conditions. Moreover, this was a single-center study performed at an obstetric HDU with personnel trained and experienced in managing critically ill pregnant and postpartum patients. As a result, our findings may not be generalizable to the general population of postpartum women in different clinical settings.

## Conclusion

Our study demonstrated that MEOWS, an early warning scale designed for pregnant and postpartum women, is more effective than pregnancy non-specific EWS in identifying postpartum women at high risk of serious complications leading to severe maternal morbidity. The results indicate that MEOWS is an accurate tool for the early recognition of postpartum clinical deterioration.

## Data Availability

Data that support the findings of the study are available from the corresponding author upon request.

## References

[1] Smith ME, Chiovaro JC, O’Neil M, Kansagara D, Quiñones AR, Freeman M (2014). Early warning system scores for clinical deterioration in hospitalized patients: a systematic review. Ann Am Thorac Soc.

[2] Burgos-Esteban A, Gea-Caballero V, Marín-Maicas P, Santillán-García A, Cordón-Hurtado MV, Marqués-Sule E (2022). Effectiveness of early warning scores for early severity assessment in outpatient emergency care: a systematic review. Front Public Health.

[3] Umar A, Ameh CA, Muriithi F, Mathai M (2019). Early warning systems in obstetrics: a systematic literature review. PLoS One.

[4] Swanton RD, Al-Rawi S, Wee MY (2009). A national survey of obstetric early warning systems in the United Kingdom. Int J Obstet Anesth.

[5] Robbins T, Shennan A, Sandall J (2019). Modified early obstetric warning scores: a promising tool but more evidence and standardization is required. Acta Obstet Gynecol Scand.

[6] Friedman AM (2015). Maternal early warning systems. Obstet Gynecol Clin North Am.

[7] Friedman AM, Campbell ML, Kline CR, Wiesner S, D’Alton ME, Shields LE (2018). Implementing obstetric early warning systems. AJP Rep.

[8] Cole FM (2014). A modified early obstetric warning system. Br J Midwifery.

[9] Hannola K, Hoppu S, Mennander S, Huhtala H, Laivuori H, Tihtonen K (2021). Obstetric early warning system to predict maternal morbidity of pre-eclampsia. Postpartum hemorrhage and infection after birth in high-risk women: a prospective cohort study. Midwifery.

[10] Edwards SE, Grobman WA, Lappen JR, Winter C, Fox R, Lenguerrand E (2015). Modified obstetric early warning scoring systems (MOEWS): validating the diagnostic performance for severe sepsis in women with chorioamnionitis. Am J Obstet Gynecol.

[11] Lappen JR, Keene M, Lore M, Grobman WA, Gossett DR (2010). Existing models fail to predict sepsis in an obstetric population with intrauterine infection. Am J Obstet Gynecol.

[12] Hedriana HL, Wiesner S, Downs BG, Pelletreau B, Shields LE (2016). Baseline assessment of a hospital-specific early warning trigger system for reducing maternal morbidity. Int J Gynecol Obstet.

[13] Shields LE, Wiesner S, Klein C, Pelletreau B, Hedriana HL (2016). Use of maternal early warning trigger tool reduces maternal morbidity. Am J Obstet Gynecol.

[14] Ryan HM, Jones MA, Payne BA, Sharma S, Hutfield AM, Lee T (2017). Validating the performance of the modified early obstetric warning system multivariable model to predict maternal intensive care unit admission. J Obstet Gynaecol Can.

[15] Nathan HL, Seed PT, Hezelgrave NL, De Greeff A, Lawley E, Anthony J (2018). Early warning system hypertension thresholds to predict adverse outcomes in pre-eclampsia: a prospective cohort study. Pregnancy Hypertens.

[16] Cavoretto PI, Rovere-Querini P, Candiani M (2022). Toward risk assessment for amniotic fluid embolisms. JAMA Netw Open.

[17] Xu Y, Zhu S, Song H, Lian X, Zeng M, He J (2022). A new modified obstetric early warning score for prognostication of severe maternal morbidity. BMC Pregnancy Childbirth.

[18] Singh S, McGlennan A, England A, Simons R (2012). A validation study of the CEMACH recommended modified early obstetric warning system (MEOWS). Anaesthesia.

[19] Singh A, Guleria K, Vaid NB, Jain S (2016). Evaluation of maternal early obstetric warning system (MEOWS chart) as a predictor of obstetric morbidity: a prospective observational study. Eur J Obstet Gynecol Reprod Biol.

[20] Yadav P, Sinha R (2023). Validating the performance of modified early obstetrics warning score (MEOWS) for prediction of obstetrics morbidity: a prospective observational study in a tertiary care institute in East India. J Obstet Gynaecol India.

[21] American College of Obstetricians and Gynecologists. (2016). Severe maternal morbidity: screening and review. obstetric care consensus No. 5. Obstet Gynecol.

[22] Shreffler J, Huecker MR. https://www.ncbi.nlm.nih.gov/books/NBK557491/.

